# Supplementary material for: The BCKDK inhibitor BT2 is a chemical uncoupler that lowers mitochondrial ROS production and de novo lipogenesis
Source: bioRxiv. 2023 Aug 16:2023.08.15.553413. Preprint. [Version 1] doi: 10.1101/2023.08.15.553413 (PMC10461965; doi:10.1101/2023.08.15.553413)
Supplement: Supplement 1 — Supplemental Table 1 – ISA modeled values and 95% confidence intervals for individual technical replicates. [file media-1.pdf]

Supplemental Table 1 – ISA modeled values and 95% confidence intervals for individual technical replicates.									
**Sample**	**Measurement**	**Value**	**Lower range**	**Upper range**	**Sample**	**Measurement**	**Value**	**Lower range**	**Upper range**
**N1-NT1**	**D(M2-AcCoA)**	0.309	0.264	0.353	**N3-NT1**	**D(M2-AcCoA)**	0.558	0.509	0.605
	**D(M1-AcCoA)**	0.027	0.006	0.052		**D(M1-AcCoA)**	0.034	0.010	0.066
	**1-D(AcCoA)**	0.665	0.621	0.708		**1-D(AcCoA)**	0.408	0.360	0.457
	**g(t) palmitate**	0.419	0.375	0.459		**g(t) palmitate**	0.456	0.408	0.498
	**D(TOTAL)**	0.335	0.270	0.405		**D(TOTAL)**	0.592	0.519	0.671
**N1-NT2**	**D(M2-AcCoA)**	0.288	0.240	0.335	**N3-NT2**	**D(M2-AcCoA)**	0.292	0.236	0.348
	**D(M1-AcCoA)**	0.027	0.006	0.055		**D(M1-AcCoA)**	0.030	0.005	0.065
	**1-D(AcCoA)**	0.685	0.638	0.731		**1-D(AcCoA)**	0.677	0.624	0.730
	**g(t) palmitate**	0.394	0.349	0.435		**g(t) palmitate**	0.354	0.307	0.396
	**D(TOTAL)**	0.315	0.246	0.390		**D(TOTAL)**	0.323	0.241	0.412
**N1-NT3**	**D(M2-AcCoA)**	0.244	0.194	0.291	**N3-NT3**	**D(M2-AcCoA)**	0.326	0.248	0.398
	**D(M1-AcCoA)**	0.032	0.009	0.064		**D(M1-AcCoA)**	0.030	0.000	0.078
	**1-D(AcCoA)**	0.724	0.678	0.769		**1-D(AcCoA)**	0.644	0.574	0.715
	**g(t) palmitate**	0.395	0.348	0.437		**g(t) palmitate**	0.262	0.211	0.306
	**D(TOTAL)**	0.276	0.204	0.355		**D(TOTAL)**	0.356	0.248	0.476
**N1-BT2-1**	**D(M2-AcCoA)**	0.322	0.255	0.385	**N3-BT2-1**	**D(M2-AcCoA)**	0.327	0.282	0.372
	**D(M1-AcCoA)**	0.029	0.000	0.070		**D(M1-AcCoA)**	0.023	0.002	0.048
	**1-D(AcCoA)**	0.650	0.588	0.712		**1-D(AcCoA)**	0.650	0.605	0.694
	**g(t) palmitate**	0.285	0.236	0.329		**g(t) palmitate**	0.366	0.325	0.403
	**D(TOTAL)**	0.350	0.255	0.455		**D(TOTAL)**	0.350	0.284	0.420
**N1-BT2-2**	**D(M2-AcCoA)**	0.299	0.221	0.370	**N3-BT2-2**	**D(M2-AcCoA)**	0.295	0.223	0.362
	**D(M1-AcCoA)**	0.030	0.000	0.079		**D(M1-AcCoA)**	0.030	0.000	0.075
	**1-D(AcCoA)**	0.671	0.603	0.739		**1-D(AcCoA)**	0.675	0.611	0.739
	**g(t) palmitate**	0.258	0.209	0.303		**g(t) palmitate**	0.235	0.191	0.274
	**D(TOTAL)**	0.329	0.221	0.449		**D(TOTAL)**	0.325	0.223	0.437
**N1-BT2-3**	**D(M2-AcCoA)**	0.231	0.076	0.320	**N3-BT2-3**	**D(M2-AcCoA)**	0.316	0.245	0.383
	**D(M1-AcCoA)**	0.046	0.004	0.169		**D(M1-AcCoA)**	0.028	0.000	0.072
	**1-D(AcCoA)**	0.723	0.646	0.826		**1-D(AcCoA)**	0.655	0.591	0.720
	**g(t) palmitate**	0.275	0.218	0.325		**g(t) palmitate**	0.248	0.202	0.289
	**D(TOTAL)**	0.277	0.079	0.489		**D(TOTAL)**	0.345	0.245	0.455
**N1-DNP-1**	**D(M2-AcCoA)**	0.355	0.236	0.455	**N3-DNP-1**	**D(M2-AcCoA)**	0.551	0.483	0.615
	**D(M1-AcCoA)**	0.024	0.000	0.095		**D(M1-AcCoA)**	0.023	0.000	0.062
	**1-D(AcCoA)**	0.621	0.524	0.721		**1-D(AcCoA)**	0.426	0.360	0.495
	**g(t) palmitate**	0.197	0.141	0.247		**g(t) palmitate**	0.111	0.088	0.134
	**D(TOTAL)**	0.379	0.236	0.550		**D(TOTAL)**	0.574	0.483	0.677
**N1-DNP-2**	**D(M2-AcCoA)**	0.387	0.243	0.505	**N3-DNP-2**	**D(M2-AcCoA)**	0.338	0.000	0.483
	**D(M1-AcCoA)**	0.022	0.000	0.105		**D(M1-AcCoA)**	0.031	0.000	0.287
	**1-D(AcCoA)**	0.591	0.476	0.713		**1-D(AcCoA)**	0.631	0.492	0.993
	**g(t) palmitate**	0.171	0.114	0.221		**g(t) palmitate**	0.175	0.110	0.231
	**D(TOTAL)**	0.409	0.243	0.610		**D(TOTAL)**	0.369	0.000	0.770
**N1-DNP-3**	**D(M2-AcCoA)**	0.346	0.201	0.456	**N3-DNP-3**	**D(M2-AcCoA)**	0.295	0.178	0.386
	**D(M1-AcCoA)**	0.025	0.000	0.109		**D(M1-AcCoA)**	0.029	0.000	0.100
	**1-D(AcCoA)**	0.629	0.524	0.743		**1-D(AcCoA)**	0.677	0.592	0.766
	**g(t) palmitate**	0.178	0.123	0.228		**g(t) palmitate**	0.172	0.127	0.212
	**D(TOTAL)**	0.371	0.201	0.565		**D(TOTAL)**	0.323	0.178	0.486
**N2-NT1**	**D(M2-AcCoA)**	0.224	0.187	0.260	**N4-NT1**	**D(M2-AcCoA)**	0.346	0.306	0.386
	**D(M1-AcCoA)**	0.031	0.012	0.054		**D(M1-AcCoA)**	0.023	0.004	0.045
	**1-D(AcCoA)**	0.746	0.711	0.780		**1-D(AcCoA)**	0.631	0.591	0.672
	**g(t) palmitate**	0.395	0.358	0.430		**g(t) palmitate**	0.378	0.340	0.412
	**D(TOTAL)**	0.254	0.198	0.314		**D(TOTAL)**	0.369	0.309	0.431
**N2-NT2**	**D(M2-AcCoA)**	0.387	0.337	0.436	**N4-NT2**	**D(M2-AcCoA)**	0.523	0.475	0.571
	**D(M1-AcCoA)**	0.024	0.002	0.052		**D(M1-AcCoA)**	0.029	0.007	0.059
	**1-D(AcCoA)**	0.589	0.540	0.638		**1-D(AcCoA)**	0.447	0.399	0.496
	**g(t) palmitate**	0.420	0.373	0.462		**g(t) palmitate**	0.429	0.383	0.470
	**D(TOTAL)**	0.411	0.339	0.488		**D(TOTAL)**	0.553	0.481	0.630
**N2-NT3**	**D(M2-AcCoA)**	0.249	0.203	0.293	**N4-NT3**	**D(M2-AcCoA)**	0.435	0.381	0.489
	**D(M1-AcCoA)**	0.030	0.008	0.059		**D(M1-AcCoA)**	0.023	0.000	0.052
	**1-D(AcCoA)**	0.721	0.679	0.763		**1-D(AcCoA)**	0.541	0.487	0.595
	**g(t) palmitate**	0.393	0.350	0.432		**g(t) palmitate**	0.412	0.363	0.455
	**D(TOTAL)**	0.279	0.212	0.352		**D(TOTAL)**	0.459	0.381	0.541
**N2-BT2-1**	**D(M2-AcCoA)**	0.398	0.295	0.492	**N4-BT2-1**	**D(M2-AcCoA)**	0.300	0.246	0.352
	**D(M1-AcCoA)**	0.029	0.000	0.094		**D(M1-AcCoA)**	0.026	0.001	0.058
	**1-D(AcCoA)**	0.573	0.480	0.669		**1-D(AcCoA)**	0.674	0.624	0.724
	**g(t) palmitate**	0.231	0.172	0.281		**g(t) palmitate**	0.266	0.226	0.302
	**D(TOTAL)**	0.427	0.295	0.586		**D(TOTAL)**	0.326	0.247	0.411
**N2-BT2-2**	**D(M2-AcCoA)**	0.282	0.203	0.354	**N4-BT2-2**	**D(M2-AcCoA)**	0.317	0.266	0.367
	**D(M1-AcCoA)**	0.032	0.000	0.084		**D(M1-AcCoA)**	0.023	0.000	0.053
	**1-D(AcCoA)**	0.685	0.619	0.753		**1-D(AcCoA)**	0.660	0.611	0.709
	**g(t) palmitate**	0.279	0.227	0.325		**g(t) palmitate**	0.232	0.198	0.263
	**D(TOTAL)**	0.315	0.203	0.437		**D(TOTAL)**	0.340	0.266	0.420
**N2-BT2-3**	**D(M2-AcCoA)**	0.369	0.271	0.459	**N4-DNP-1**	**D(M2-AcCoA)**	0.365	0.247	0.468
	**D(M1-AcCoA)**	0.030	0.000	0.091		**D(M1-AcCoA)**	0.026	0.000	0.093
	**1-D(AcCoA)**	0.601	0.514	0.690		**1-D(AcCoA)**	0.609	0.508	0.714
	**g(t) palmitate**	0.252	0.193	0.303		**g(t) palmitate**	0.183	0.133	0.229
	**D(TOTAL)**	0.399	0.271	0.549		**D(TOTAL)**	0.391	0.247	0.561
**N2-DNP-1**	**D(M2-AcCoA)**	0.464	0.319	0.590	**N4-DNP-2**	**D(M2-AcCoA)**	0.340	0.271	0.408
	**D(M1-AcCoA)**	0.021	0.000	0.117		**D(M1-AcCoA)**	0.019	0.000	0.056
	**1-D(AcCoA)**	0.515	0.390	0.650		**1-D(AcCoA)**	0.641	0.576	0.706
	**g(t) palmitate**	0.150	0.094	0.201		**g(t) palmitate**	0.163	0.129	0.195
	**D(TOTAL)**	0.485	0.319	0.707		**D(TOTAL)**	0.359	0.271	0.464
					**N4-DNP-3**	**D(M2-AcCoA)**	0.316	0.239	0.388
						**D(M1-AcCoA)**	0.021	0.000	0.064
						**1-D(AcCoA)**	0.663	0.594	0.732
						**g(t) palmitate**	0.162	0.126	0.194
						**D(TOTAL)**	0.337	0.239	0.452
